# Expression and shedding of endothelial protein C receptor in prostate cancer cells

**DOI:** 10.1186/1475-2867-11-4

**Published:** 2011-02-15

**Authors:** Mario Menschikowski, Albert Hagelgans, Oliver Tiebel, Ludwig Klinsmann, Graeme Eisenhofer, Gabriele Siegert

**Affiliations:** 1 Institute of Clinical Chemistry and Laboratory Medicine, Technical University of Dresden, Medical Faculty "Carl Gustav Carus", Fetscherstrasse 74, D-01307 Dresden, Germany

## Abstract

**Background:**

Increasing evidences show that beyond its role in coagulation, endothelial protein C receptor (EPCR) interferes with carcinogenesis. Pro-carcinogenic effects of EPCR were linked with a raised generation of activated protein C (aPC) and anti-apoptotic signalling. This study was carried out to analyze the expression, cell surface exposition, and shedding of EPCR in normal and malignant prostate cell lines.

**Results:**

EPCR expression is up-regulated both at the mRNA and protein levels in invasive prostate DU-145 and PC-3 cells in comparison to normal prostate epithelial cells (PrEC) and less-invasive LNCaP cells. Release of soluble EPCR (sEPCR) is induced by 12-myristate 13-acetate, ionomycin, H_2_O_2_, and disruptor of lipid rafts in PrEC, DU-145, and PC-3 cells. Furthermore, interleukin-1β (IL-1β) and tumor necrosis factor-α (TNF-α), but not interleukin-6 or interferon-γ increase sEPCR release. In LNCaP cells, neither pharmacological agents nor IL-1β or TNF-α result in a significant increase of sEPCR release. The effects of IL-1β and TNF-α on EPCR shedding in DU-145 cells are mediated by MEK/ERK 1/2, JNK, and p38 MAPK signalling cascades. In PC-3 cells, however, the MEK/ERK 1/2 pathway is down-regulated and incubation with cytokines did not elevate the phosphorylated ERK-1/2 fraction as in the case of DU-145 cells. Treatment with 4-aminophenylmercuric acetate (APMA), an activator of metalloproteases, causes a disproportionately large increase of sEPCR release in DU-145 and PC-3 cells, compared to PrEC and LNCaP cells. Finally, an increased release of sEPCR mediated by APMA treatment is shown to be connected with reduced generation of activated protein C indicating the functionality of EPCR in these cells.

**Conclusions:**

The study demonstrates a number of substantial differences in expression and shedding of EPCR in prostate cancer cell lines in comparison with normal cells that may be relevant for understanding the role of this receptor in carcinogenesis.

## Background

Prostate cancer remains one of the most common forms of cancer affecting men today [1]. Patients with metastatic hormone-refractory prostate carcinoma often have dramatic and life-threatening coagulation complications from their disease characterized by both induced coagulation and bleeding diathesis [2,3]. Frequently, disseminated intravascular coagulation is a complication in prostate cancer patients. Additional coagulopathies in these patients are thrombocytopenic thrombotic purpura, thrombosis, Trousseau's syndrome, and acquired factor VIII inhibitor development [2]. A causal link between cancer and thrombosis seems related to abnormally high levels of coagulation factors and reduced levels of natural anticoagulants [4,5]. In cancer patients there is a constantly up-regulated generation of thrombin with potential procarcinogenic actions that can be counteracted by anticoagulant and anti-inflammatory protein C/thrombomodulin-mediated mechanisms [6-8]. Such relationships provide a rationale for studies of the activity and function of the anticoagulant protein C (PC) pathway in malignancy and metastasis [8-10]. This PC-pathway includes as key components the thrombin-thrombomodulin complex and the endothelial protein C receptor (EPCR) acting as a co-receptor [11].

Activated protein C (aPC) has divergent effects on tumour cell migration, invasion, and metastasis. On the one hand, aPC acts as a pro-carcinogenic agent by way of EPCR- and PAR-1-mediated survival- and anti-apoptotic signalling pathways [12-14]. On the other hand, aPC may exert anti-metastatic effects by inhibiting cancer cell adhesion, extravasation, and cancer cell-induced vascular leakage [15,16]. Both expression and functional activity of EPCR in prostate cancer cells are still unknown. In view of convincing evidence that EPCR, beyond its effects on coagulation, interferes with carcinogenesis, the present study was carried out to analyze the expression, cell surface exposition, and shedding of this receptor in normal and malignant prostate cell lines.

## Results

### Differential expression of endothelial protein C receptor in prostate cancer cells

**Figure 1:**
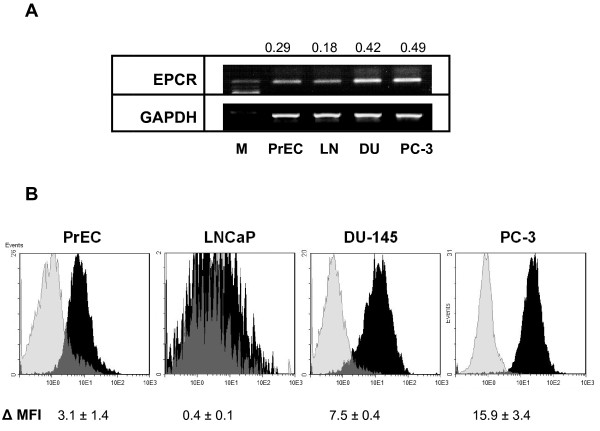
**Expression of endothelial protein C receptor in normal and malignant prostate cells**. (A) Agarose gel electrophoresis with ethidium bromide-stained amplificates of EPCR and GAPDH-specific mRNA in normal prostate epithelial cells and prostate cancer cell lines. M, 100 bp molecular weight ladder; PrEC, prostate epithelial cells; LN, LNCaP cells; and DU, DU-145 cells. Numbers at the top of the images represent the ratio of EPCR/GAPDH mRNA calculated from the densitometry values of amplificates. Data are representative of three independent experiments giving similar results. (B) Flow cytometry analysis of EPCR immunoreactivities in prostate cells. Cells were incubated with anti-EPCR RCR-252 monoclonal antibodies followed by incubation with FITC-conjugated secondary antibodies and fixation of cells. Black shading represents tracks obtained with specific primary antibodies; gray shading shows control cells treated with corresponding isotype IgG. Values at the bottom of images represent the mean fluorescence intensities (MFI) corrected for values in related controls. Results are the means ± SDs of analyses in duplicates obtained in three similar experiments.

In comparison to normal human prostate epithelial cells (PrEC), levels of EPCR-specific mRNA were higher in PC-3 and DU-145 cells and lower in LNCaP cells (Figure 1A). At protein levels, similar results were indicated by flow cytometry analyses using anti-EPCR monoclonal antibody (Figure 1B). In DU-145 and PC-3 cell lines, EPCR-specific immunofluorescence signals were 2.4- and 5.1-fold higher in comparison to normal cells (PrEC), whereas the EPCR signals in LNCaP cells were negligible.

**Figure 2:**
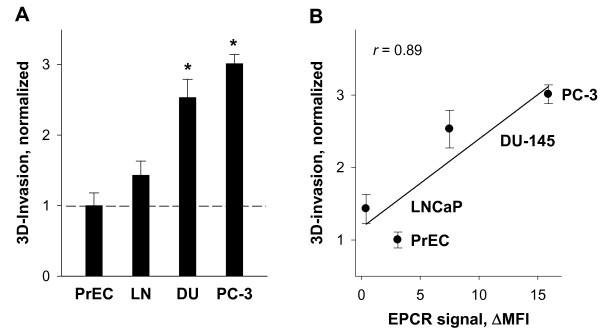
**Invasiveness of prostate cancer cells (A) and its correlation with EPCR expression (B)**. (A) Data bars represent the relative invasion activity of normal PrEC and malignant LNCaP (LN), DU-145 (DU), and PC-3 prostate cell lines measured in 3D cell invasion assay (Oris™). Serum starved cells were seeded onto basement matrix extract coated plates. Following 48 hr incubation, cells were labeled with Calcein AM and the fluorescence in the analytic zone was quantified using a plate reader, Victor3 1420 Multilabel Counter. Data are expressed as changes relative to PrEC cell migration which was assigned a value of 1.0. Results are the means ± SDs of analyses in quadruplicates and are representative of two independent experiments (B) Correlation of invasion activity and EPCR-specific immunostainings in PrEC and prostate cancer cell lines. Correlation coefficient was calculated using SigmaPlot 11 (Systat software, Inc.).

To examine possible relationships between invasive potential and levels of EPCR in cancer cells, we next studied the invasion activity of normal and malignant prostate cells. For this purpose, an Oris™ novel fluorescence assay was utilized to monitor movement of cells through detection zones in a 3-D extracellular matrix. This analysis indicated distinctly higher invasion of PC-3 and DU-145 cells into 3D matrix than invasion of LNCaP cells and PrEC (Figure 2A). A positive relationship (*r *= 0.89) was observed between levels of cell surface EPCR (flow cytometry data) and numbers of invading cells within the 3D matrix (Figure 2B).

### Pharmacological and physiological inducers of EPCR shedding in prostate cells

**Figure 3:**
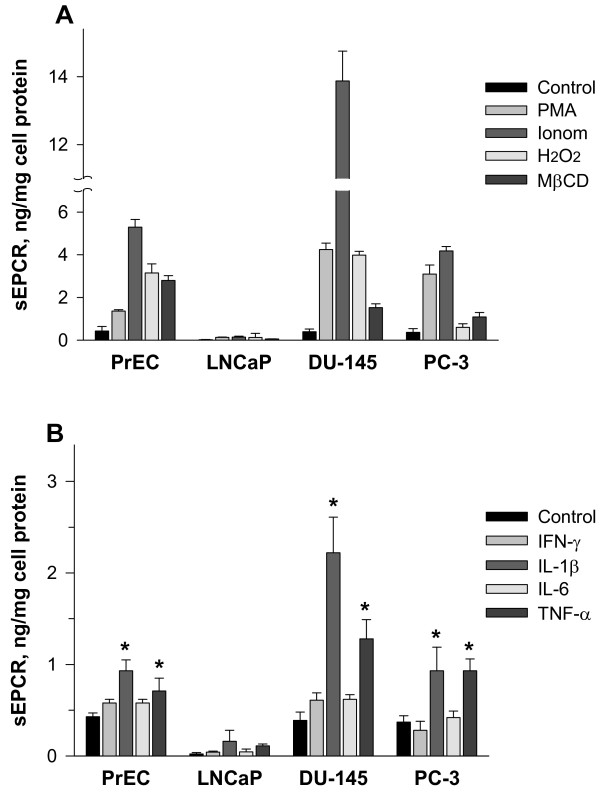
**Stimulation of sEPCR release in normal PrEC and prostate cancer cell lines by pharmacological agents and pro-inflammatory cytokines**. Cells were incubated for 1 h either with 100 ng/ml PMA, 3 μM ionomycin, 0.8 mM H_2_O_2_, and 4 mM MβCD (A) or with pro-inflammatory cytokines (IFN-γ, IL-1β, IL-6, and TNF-α) at final concentrations of 25 ng/ml (B). Released sEPCR in cell culture medium was determined using sEPCR ELISA technique. Results are the means ± SDs of analyses in triplicates and are representative of two similar experiments. * p < 0.05 *versus *controls without cytokines.

In normal PrEC as well as DU-145 and PC-3 cells, the release of sEPCR was stimulated by pharmacological inducers such as phorbol-12-myristate 13-acetate (PMA), ionomycin, H_2_O_2_, and methyl-β-cyclodextrin (MβCD) (Figure 3A). The most prominent effect was observed for ionomycin in DU-145, which caused a much more pronounced induction of the release of sEPCR in DU-145 compared with PrEC and PC-3 cells. In LNCaP cells, all these pharmacological agents were ineffective at inducing sEPCR release. Furthermore, treatment of cells with MβCD as a cholesterol-depleting agent stimulated the release of sEPCR (Figure 3A). Interestingly, the effect of MβCD on sEPCR release was highest in normal PrEC, whereas in malignant prostate cell lines the stimulatory effect was distinctly less (Figure 3A).

The pro-inflammatory cytokines, including IL-1β, IL-6, TNF-α, and IFN-γ, had variable influences on EPCR shedding in prostate cells (Figure 3B). In normal PrEC as well as malignant DU-145 and PC-3 cells, IL-1β and TNF-α significantly increased sEPCR release, with the most pronounced effects observed in DU-145 cells; in contrast, IFN-γ and IL-6 were almost ineffective. In LNCaP cells, IL-1β and TNF-α had insignificant effects on sEPCR release (Figure 3B).

**Figure 4:**
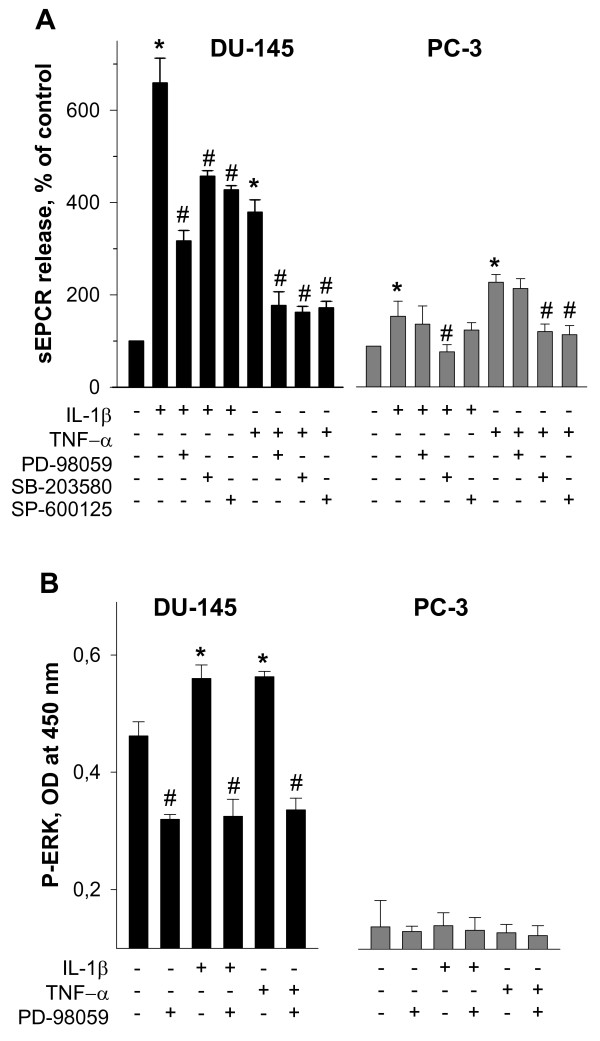
**Effects of MAPK signaling inhibitors on cytokine-induced EPCR shedding (A) and activation of ERK 1/2 pathway (B) in DU-145 and PC-3 prostate cancer cell lines**. (A) Cells were pre-incubated with 50 μM PD-98059, 10 μM SB-203580, or 20 μM SP-600125 as indicated for 30 min and thereafter exposed to cytokines at a final concentration of 25 ng/ml for additional 5 h. Results are the means ± SDs of analyses in triplicates and are representative of three independent experiments. * *p *< 0.05 *versus *control without cytokines; # *p *< 0.05 *versus *cells exposed to cytokines alone. (B) DU-145 and PC-3 prostate cancer cells were incubated with cytokines at a final concentration of 25 ng/ml and 50 μM PD-98059 as indicated for 10 min. Signals of total and phosphorylated forms of ERK 1/2 (P-ERK 1/2) were determined using cell-based ELISA kit. OD, optical density. Results are the means ± SDs of analyses in triplicates and are representative of two similar experiments. * *p *< 0.05 *versus *control without cytokines and PD-98059; # *p *< 0.05 *versus *cells treated with cytokines, but not with PD-98059.

A panel of pharmacological inhibitors of MAP kinases produced variable effects on IL-1β- and TNF-α-induced shedding of EPCR in DU-145 and PC-3 prostate cancer cells (Figure 4A). Distinct attenuation of sEPCR release in DU-145 cells was observed after treatments with PD-98059 as pharmacological inhibitor of MEK/ERK 1/2 activity, SB-203580 as inhibitor of p38 MAPK, and SP-600125 as inhibitor of c-Jun N-terminal kinase (JNK) MAPK. In PC-3 cells, PD-98059 was effective at abolishing both the IL-1β- and the TNF-α-induced release of sEPCR, whereas SP-600125 significantly attenuated TNF-α-induced but not IL-1β-induced shedding of EPCR.

To confirm the conclusion that in PC-3 cells the MEK/ERK 1/2 pathway is down-regulated, levels of ERK 1/2 phosphorylation in DU-145 and PC-3 cells were measured using a cell-based ELISA assay. Under normal conditions ERK 1/2 was more higher phosphorylated in DU-145 cells than in PC-3 cells (Figure 4B). Exposure of DU-145 cells, but not of PC-3 cells, to IL-1β and TNF-α led to a further increase of phosphorylated ERK 1/2 which was attenuated by treatment with PD-98059. Therefore, these data suggest that the MEK/ERK 1/2 pathway in PC-3 cells is down-regulated.

### Involvement of metalloproteases in shedding of EPCR in prostate cells

**Figure 5:**
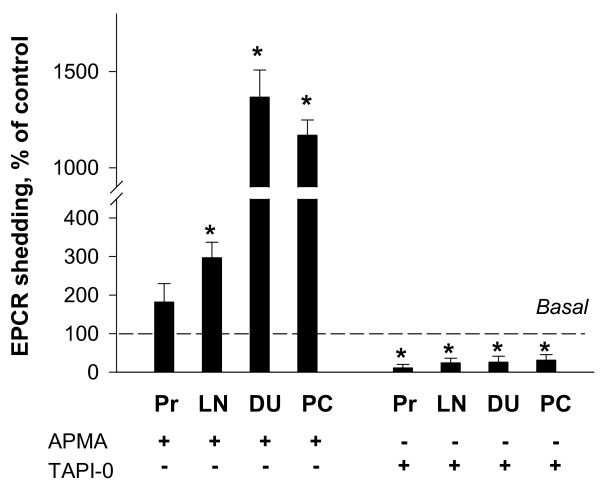
**Effects of modulation of metalloprotease activities on sEPCR release in prostate cells**. Bars show the effects of metalloprotease inhibitor, 30 μM TAPI-0, and metalloprotease activator, 60 μM APMA, on shedding of EPCR in prostate cells. Released sEPCR was measured in culture medium of cells treated with and without TAPI-0 and APMA as indicated using ELISA technique. Pr, prostate epithelial cells; LN, LNCaP cells; DU, DU-145 cells; and PC, PC-3 cells, respectively. The release of sEPCR in control (without TAPI-0 and APMA treatment) was assigned as 100%. Results are the means ± SDs of analyses in triplicates and are representative of three independent experiments. * p < 0.05 versus control without TAPI-0 or APMA.

The effects of 4-aminophenylmercuric acetate (APMA), a generic organomercurial activator of metalloproteases, and TAPI-0, a broad spectrum protease inhibitor, indicated involvement of metalloproteases in shedding of EPCR (Figure 5). More specifically, treatment of cells with APMA resulted in a disproportionally larger increase (by about 14- and 12-fold) of released sEPCR levels in DU-145 and PC-3 cells than in untreated cells. In LNCaP cells, treatment with APMA led also to a significant, but less pronounced increase (by about 2.6-fold) of sEPCR release. In PrEC the effect of APMA on sEPCR release did not differ significantly compared to untreated cells (Figure 5). Conversely, the release of sEPCR was significantly attenuated by 30 μM TAPI-0 in all analyzed cell lines (Figure 5).

**Figure 6:**
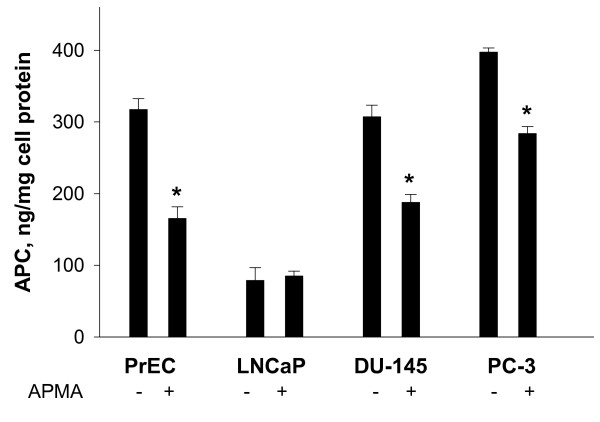
**Effects of APMA as metalloproteinase activator on thrombin-dependent activation of protein C in prostate cells**. Levels of aPC were determined after a 2 hr-incubation period in prostate cells without or with 60 μM APMA exposure. Subsequently, cells were washed and further incubated for 1 h in the presence of 4 μg/ml protein C and 0.12 NIH U thrombin/well. Aliquots of supernatants were transferred into 96-well plates and assayed for aPC generation using chromogenic substrate S-2366. Results are the means ± SDs of analyses in triplicates and are representative of two independent experiments. * p < 0.05 versus controls without APMA.

In order to assess the functionality of EPCR expressed in prostate cells, the activation of protein C in dependence on APMA-induced EPCR shedding was studied. In non-cancerous PrEC as well as in cancerous DU-145 and PC-3 cells nearly similar levels of aPC were produced from exogenously added protein C (Figure 6). In LNCaP cells, weak expression of EPCR correlated with a small generation of aPC. After induction of sEPCR release by APMA the levels of generated aPC significantly decreased in PrEC, DU-145 and PC-3 cells. There was no evident APMA-mediated effect on protein C activation in LNCaP cells (Figure 6).

## Discussion

The present study elucidates novel findings concerning the expression and shedding of EPCR in normal epithelial prostate cells (PrEC) and malignant LNCaP, DU-145, and PC-3 prostate cell lines. In particular, our data establishes that EPCR is up-regulated in DU-145 and PC-3 cell lines compared to normal PrEC or less invasive LNCaP malignant cells and that the expression of EPCR correlates with the invasiveness of the various prostate cell lines. Similar up-regulation of EPCR was previously described in different primary malignant cells and cell lines derived from tumours generally considered to be poorly responsive to chemotherapy, such as colon carcinomas, renal cell carcinomas, and melanomas [9,17]. Increasing evidence suggests that an up-regulation of EPCR may contribute to tumor progression, but the underlying mechanisms remain obscure [17,18]. Pro-carcinogenic effects of EPCR are suggested to be linked with the stimulation of aPC generation and anti-apoptotic signaling [19]. Given the function of aPC as an anticoagulant, it was hypothesized that EPCR expression by tumour cells provides a growth advantage by maintaining a coagulation-free environment *in vivo *[17]. Binding of aPC to corresponding receptors, EPCR and PAR-1, leads also to a stimulation of cell motility, invasion, and angiogenesis [13,20]. In addition, aPC may affect cellular invasion either *via *direct activation of matrix metalloproteases or by binding to plasminogen activator inhibitor-1 leading to activation of extracellular matrix proteases and thereby increasing cellular invasion [21,22].

Despite of these pro-carcinogenic effects, an inhibitory role of the aPC/EPCR pathway on tumor-endothelium interactions has been described recently [15]. This anti-metastatic effect of therapeutic doses of aPC is realized *via *inhibition of tumor cell adhesion and transmigration. Further evidence suggests that endogenous aPC limits cancer cell extravasation and cancer cell-induced vascular leakage in a sphingosine-1-phosphate receptor-1 dependent manner [16]. In view of these findings it is of great interest to understand in detail the mechanisms regulating the exposition of EPCR on the malignant cell surface. First, previous studies demonstrated that levels of EPCR exposed at endothelial cell surfaces are markedly changed by its ectodomain cleavage and release in soluble form (sEPCR) [23-25]. Second, released sEPCR promotes an increased tendency for coagulation, probably through competition for aPC/PC [17,26]. In line with these activities, increased levels of sEPCR may interfere with the above-mentioned anti-metastatic effects of aPC.

Similar to previous observations in HUVEC [25], the shedding of EPCR in normal PrEC and malignant prostate DU-145 and PC-3 cell lines is induced by PMA, ionomycin, and H_2_O_2_. Furthermore, MβCD as a disruptor of lipid rafts increases the release of sEPCR in these cells. The observation that the effect of MβCD on sEPCR release is lower in DU-145 and PC-3 cells in comparison to normal PrEC can be explained by elevated cholesterol levels in cancer cells compared to non-cancerous cells, increased amounts of lipid rafts and reduced sensitivity to decreased cholesterol levels [27,28].

In addition to these pharmacological agents, pro-inflammatory cytokines such IL-1β and TNF-α, but not INF-γ and IL-6 induce the release of sEPCR in PrEC, DU-145, and PC-3 cells. These data agree with previous observations in HUVEC [25]. However, in LNCaP cells neither pharmacological agents nor IL-1β and TNF-α led to increased release of sEPCR. Herein a further specific feature exists in this cell line that agrees with other already described clear differences between LNCaP and androgen-independent cell lines, DU-145 and PC-3, in terms of their regulation of the phosphatidylinositol 3-kinase (PI3K)/protein kinase B (PKB/Akt), Jak/STAT3, JNK MAPK and other signaling pathways [29-31].

In order to further elucidate the mechanisms responsible for the IL-1β and TNF-α-mediated increase of sEPCR release in invasive prostate cell lines we investigated the involvement of MAPK pathways using selective inhibitors. Previously we described that in HUVEC the cytokine-induced shedding of EPCR is mediated by down-stream MAP kinases including ERK 1/2, JNK, and p38 pathways [25]. Here, it is shown that the effects of IL-1β and TNF-α on EPCR shedding in DU-145 cells are also mediated by ERK 1/2, JNK, and p38 MAPK signalling cascades. In PC-3 cells, however, the MEK/ERK 1/2 pathway is down-regulated. This is supported further by the observation that PD-98059 failed to attenuate the cytokine-induced sEPCR release and that IL-1β and TNF-α did not elevate the phosphorylated MEK/ERK-1/2 fraction in these cells. This finding may also explain the limited effects of IL-1β, TNF-α, and ionomycin on sEPCR release in PC-3 compared to DU-145 cells. Moreover, numerous cellular effects of hydrogen peroxide are known to be mediated by aberrant activation of the MEK/ERK-1/2 pathway [32]. This may explain the low efficiency of H_2_O_2 _as shedding inducer in PC-3 in comparison to DU-145 cells (Figure 3A).

This study also shows that shedding of EPCR is disproportionally induced by the metalloprotease activator, APMA, in invasive DU-145 and PC-3 cells compared to PrEC and less invasive LNCaP cells. This difference can be explained by the well-known up-regulation of metalloproteases in DU-145 and PC-3 cells than in normal or less invasive prostate cells [33]. The importance of metalloproteases in EPCR shedding is shown also by the complete inhibition of sEPCR release through the broad-spectrum inhibitor of metalloproteases, TAPI-0, in all analyzed cell lines.

To elucidate the functionality of EPCR in normal and malignant prostate cells we addressed two questions: (i) whether exogenous protein C is converted into aPC in PrEC and malignant prostate cells and (ii) whether the generation of aPC is modified by treatment of cells with APMA. Our finding that generation of aPC in DU-145 and PC-3 cell lines is similar to PrEC and that it is significantly diminished after APMA-mediated induction of EPCR shedding suggests that the protein C pathway in PrEC, DU-145, and PC-3 is functionally active and that increased levels of EPCR at DU-145 and PC-3 cell surfaces may contribute to effective aPC production. In LNCaP cells, the low protein C activation correlates with down-regulated expression of EPCR.

## Conclusions

This study brings to light new information concerning expression and shedding of EPCR in normal and malignant human prostate cell lines. The demonstrated up-regulation of EPCR in invasive cancer cell lines may provide a potential biological marker for prostate malignancies. Cell surface levels of EPCR can effectively be changed by activation or inhibition of cell signaling cascades and metalloproteases involved in its shedding. The present elucidation of mechanisms underlying the regulation of expression and proteolytic cleavage of EPCR suggests new potential avenues to interfere with tumour growth and malignancy.

## Materials and methods

### Materials

Phorbol-12-myristate 13-acetate, PD-98059, SB-203580, SP-600125, TAPI-0, ionomycin, thrombin, and recombinant hirudin were from Calbiochem (Schwalbach, Germany). Human plasma-derived PC and aPC were from Hematological Technologies Inc. (Cell Systems, Biotechnologie Vertrieb GmbH, St. Katharinen, Germany). Calcein AM, bovine serum albumin (BSA), 4-aminophenylmercuric acetate, and methyl-β-cyclodextrin were purchased from Sigma-Aldrich (Deisenhofen, Germany). Recombinant human IL-1β, TNF-α, IFN-γ, and IL-6 were from Roche Diagnostics GmbH (Mannheim, Germany), chromogenic substrate S-2366 from Haemochrom Diagnostica GmbH (Essen, Germany). Calcein AM, PMA, PD-98059, SB-203580, SP-600125, TAPI-0, ionomycin, and APMA were dissolved in dimethyl sulfoxide (DMSO). The final concentrations of DMSO were 0.3% or less, and controls using DMSO alone were run in all cases. Other agents were used as aqueous solutions. Monoclonal anti-EPCR antibody produced in rat, RCR-252, and anti-rat IgG-FITC conjugated antibody produced in goat were from Sigma-Aldrich (Deisenhofen, Germany).

### Cell culture and incubation

Normal human prostate epithelial cells (PrEC; Cambrex Bio Science, Walkersville, MD, USA) were maintained up to a maximum of six passages in prostate epithelial growth medium supplemented with bovine pituitary extract, epidermal growth factor, insulin, transferrin, hydrocortisone, retinoic acid, epinephrine, triiodothyronine and gentamicin-amphotericin solution, according to the manufacturer's instruction. Every two to three days the medium was changed and before reaching confluence, cells were passaged using trypsin/EDTA. Human prostate malignant cell lines (PC-3, DU-145, and LNCaP cells) were purchased from German Collection of Microorganisms and Cell Cultures (Berlin, Germany). They were cultured in standard cell culture medium RPMI 1640 supplemented with 10% heat-inactivated fetal calf serum (FCS), 2 mM L-glutamine, 100 U/ml penicillin, and 100 μg/ml streptomycin at 37°C in a humidified atmosphere of 5% CO_2_.

### RNA Extraction and RT-PCR Analysis

RNA was isolated after lysis of cells in TRI Reagent according to the manufacturer`s instructions. Isolated RNA was converted to cDNA using the GeneAmp RNA-PCR Kit (PerkinElmer LAS GmbH, Jügesheim, Germany). A portion of the RT reaction products was then amplified for identification of EPCR- and glyceraldehyde-3-phosphate dehydrogenase (GAPDH)-specific mRNA as a reference gene using PCR. Early described primers were used to amplify the coding sequences of human EPCR: 5'-TGG CCT TTC CTC TGA CCA TCC-3` (sense) and 5'-GGA GCT CCC ATT CAC AGC CAC-3` (antisense) giving PCR products with a length of 100 bp [34]. The further applied primer pair was 5`-CGG AGT CAA CGG ATT TGG TCG TAT TG-3` and 5`-GCA GGA GGC ATT GCT GAT GAT CTT G-3` for GAPDH amplifying products with 439 bp length. Primer pairs were applied in a final concentration of 0.8 μM. The buffers and reagents used were from GeneAmp Kit (PerkinElmer LAS GmbH). After amplification, the PCR products were subjected to agarose gel electroforesis and photographed using a G:BOX Chemi, GelVue UV Transilluminator devise (SynGene, USA). Images were analysed using GeneTools software from SynGene.

### Flow cytometry

After incubation cells were scraped off the culture dishes, washed in phosphate buffered saline (PBS), pH 7.4, and resuspended at 1 × 10^6 ^cells/ml in FACS buffer (PBS supplemented with 1% BSA and 0.1% sodium azide). Cells were then incubated with anti-EPCR rat monoclonal antibody RCR-252 added to a final concentration of 2.5 μg/ml for 30 min at 4°C. Subsequently, cells were washed twice with FACS-buffer and incubated under light protected conditions for 30 min at 4°C with FITC-conjugated anti-rat secondary antibodies, which were added to a final dilution of 1:100 of the commercially supplied stock solution. Finally, cells were again washed twice, fixed in 4% paraformaldehyde in PBS, and analyzed on EPICS XL flow cytometer (Beckman Coulter GmbH, Krefeld, Germany). Isotype rat IgG was used instead of primary antibodies as controls for EPCR determination.

### ELISA based quantitative determination of sEPCR

Amounts of sEPCR released by prostate cells were determined using Asserachrom sEPCR ELISA kits (Diagnostica Stago, Asnieres, France) according to the manufacturer`s instructions. For this purpose, cells were grown to confluence in 96-well microplate in complete medium. After this, the medium was refreshed and cells were further incubated with inducers or inhibitors of EPCR shedding. At the end of incubations, medium was removed, centrifuged at 800 g for 10 min to remove the cell debris and used for analysis without further dilution to determine sEPCR levels released by cells. Total cell protein was determined using a Bicinchoninic Acid assay kit (Sigma-Aldrich, Deisenhofen, Germany) with bovine serum albumin as internal standard.

### Determination of ERK 1/2 phosphorylation with cell-based ELISA

Prostate cancer cells were cultured in 96-well microplates for quantitative determination of ERK 1/2 phosphorylation. On the day of experiments, culture medium was replaced by serum-free growth medium. After a 30-minute pre-incubation period with or without 50 μM PD-98059 as a selective inhibitor of the MEK/ERK pathway, either 25 ng/ml IL-1β or 25 ng/ml TNF-α was added directly into wells and cells were further incubated for set periods of time. Levels of total ERK and phosphorylated ERK (P-ERK) were quantified in fixed cells using a RayBio Cell-based P-ERK 1/2 (Thr202/Tyr204) ELISA kit (BioCat GmbH, Heidelberg, Germany) according to the manufacturer's instructions.

### Prostate cancer cell 3D invasion assay

Cell invasion was measured *in vitro *using Oris™ Cell Invasion & Detection Assay (AMS Biotechnology Ltd, Abingdon OX14 4SE, UK) according to the manufacturer`s instructions. Briefly, after serum starvation for 18 hr cells (50,000 cells/well) were seeded on the Oris™ BME (Basement Membrane Extract) coated microplate and allowed to adhere overnight. Stoppers were removed and cells were overlaid with BME in the presence of 10% FBS. After a 48-hr incubation, cells were stained with Calcein AM reagent. The detection mask was applied to the bottom of the microplate and fluorescence from cells in the detection zone was quantified using a Victor3 1420 Multilabel Counter reader (PerkinElmer LAS GmbH, Rodgau Jügesheim, Germany) at excitation/emission wavelengths of 485/520 nm.

### Protein C activation assay

Cells were cultured in 24-well plates, treated as indicated and subsequently washed three times in buffer A containing 50 mM Tris-HCI (pH 7.5), 2 mM CaCl_2_, 100 mM NaCl, and 0.1% BSA. Washed cells were incubated for 2 h at 37°C in the presence of human protein C (4 μg/ml), thrombin (0.12 NIH U pro well), and buffer A in a final volume of 200 μl/well. Thereafter, 150 μl of supernatants were transferred into 96-well plates and assayed for the generation of aPC using 0.8 mM chromogenic substrate S-2366. To prevent nonspecific cleavages of S-2366 by thrombin, hirudin (10 antithrombin units pro well) was added to each probe. Extinction of reaction product was measured at 405 nm on Victor3 1420 Multilabel Counter reader. Amounts of generated aPC were calculated using aPC standards and normalized to cell protein content.

### Data analysis

Data were analyzed by one-way analysis of variance coupled with Dunnett's *post hoc *test to compare each experimental group with a nominated control group using SPSS 14.0 software. Differences were considered significant at *P *< 0.05.

## List of abbreviations

aPC: activated protein C; APMA: 4-aminophenylmercuric acetate; BSA: bovine serum albumin; DMSO: dimethyl sulfoxide; EPCR: endothelial protein C receptor; ERK ½: extracellular signal-regulated kinase; FCS: fetal calf serum; HUVEC: human umbilical vein endothelial cells; IFN-γ: interferon-γ; IL-1β: interleukin-1β; IL-6: interleukin-6; JNK: c-Jun N-terminal kinase; MAPK: mitogen-activated protein kinase; MβCD: methyl-β-cyclodextrin; MEK: mitogen-activated/ERK kinase; MMP: matrix metalloproteinase; PBS: phosphate-buffered saline; PC: protein C; PKC: protein kinase C; PMA: phorbol 12-myristate 13-acetate; ROS: reactive oxygen species; sEPCR: soluble endothelial protein C receptor; TM: thrombomodulin; TNF-α: tumor necrosis factor-α.

## Competing interests

The authors declare that they have no competing interests.

## Authors' contributions

MM and AH conceived and designed the experiments and drafted the manuscript. OT and LK performed the flow cytometry analysis. GS and GE coordinated the study and edited the manuscript. All authors have read and approved the final manuscript.
